# Health Effects of Phenolic Compounds Found in Extra-Virgin Olive Oil, By-Products, and Leaf of *Olea europaea* L.

**DOI:** 10.3390/nu11081776

**Published:** 2019-08-01

**Authors:** Annalisa Romani, Francesca Ieri, Silvia Urciuoli, Annalisa Noce, Giulia Marrone, Chiara Nediani, Roberta Bernini

**Affiliations:** 1 PHYTOLAB (Pharmaceutical, Cosmetic, Food Supplement, Technology and Analysis)-DiSIA, University of Florence, Via U. Schiff, 6, 50019 Sesto Fiorentino, Italy; 2 UOC of Internal Medicine-Center of Hypertension and Nephrology Unit, Department of Systems Medicine, University of Rome Tor Vergata, Via Montpellier 1, 00133 Rome, Italy; 3 PhD School of Applied Medical, Surgical Sciences, University of Rome Tor Vergata, via Montpellier 1, 00133 Rome, Italy; 4 Department of Experimental and Clinical Biomedical Sciences “Mario Serio”, University of Florence, Viale Morgagni 50, 50134 Florence, Italy; 5 Department of Agriculture and Forest Sciences (DAFNE), University of Tuscia, Via San Camillo de Lellis, 01100 Viterbo, Italy

**Keywords:** *Olea europaea* L., extra-virgin olive oil, olive oil by-products, olives leaf, phenolic compounds, hydroxytyrosol, oleuropein, oleocanthal, lignans, health effects, circular economy

## Abstract

*Olea europaea* L. fruit is a peculiar vegetal matrix containing high levels of fatty acids (98–99% of the total weight of extra-virgin olive oil, EVOO) and low quantities (1–2%) of phenolics, phytosterols, tocopherols, and squalene. Among these minor components, phenolics are relevant molecules for human health. This review is focused on their beneficial activity, in particular of hydroxytyrosol (HT), oleuropein (OLE), oleocanthal (OLC), and lignans found in EVOO, olive oil by-products and leaves. Specifically, the cardioprotective properties of the Mediterranean diet (MD) related to olive oil consumption, and the biological activities of polyphenols recovered from olive oil by-products and leaves were described. Recent European projects such as EPIC (European Prospective Investigation into Cancer and Nutrition) and EPICOR (long-term follow-up of antithrombotic management patterns in acute coronary syndrome patients) have demonstrated the functional and preventive activities of EVOO showing the relation both between cancer and nutrition and between consumption of EVOO, vegetables, and fruit and the incidence of coronary heart disease. The data reported in this review demonstrate that EVOO, one of the pillars of the MD, is the main product of *Olea europaea* L. fruits; leaves and by-products are secondary but precious products from which bioactive compounds can be recovered by green technologies and reused for food, agronomic, nutraceutical, and biomedical applications according to the circular economy strategy.

## 1. Introduction

*Olea europaea* L. is a fruit tree native to Asia Minor and Syria, which today is cultivated in the entire Mediterranean area; nowadays, the major producers of olives and olive oil are Spain, Italy, and Greece. Extra-virgin olive oil (EVOO), extracted physically from the fruit, is known for its nutritional properties and health effects, especially against cardiovascular diseases (CVDs). These properties are due to the presence of high levels of fatty acids (98–99% of the total weight of EVOO), in particular of monounsaturated acids such as oleic, as well as of other valuable components like phenolics, phytosterols, tocopherols, and squalene even if present in low percentages (1–2%). Only EVOO, and not all seed oils, has a high percentage of fatty acids with a correct balance of unsaturated fatty acids stabilized by minor polar compounds, with an antioxidant character [1].

All components of EVOO may potentially contribute to “health maintenance” [2]. Several international organisms regulate the quality and purity of EVOO, namely the European Union (EU), the International Olive Council (IOC), and the Codex Alimentarius according to data compiled by the Unaprol Economic Observatory [3].

Tree cultivars, their growing conditions, and the techniques used for EVOO production, are key factors for the quality of EVOO, affecting both its qualitative and quantitative characteristics, which can influence the sensorial and health properties of the oil. Advanced analytical techniques, such as high-performance liquid chromatography coupled to a diode array or a mass detector (HPLC-DAD, HPLC-MS), have played an important role in identifying and quantifying the bioactive compounds found in EVOO, which are responsible for its beneficial effects.

**Figure 1:**
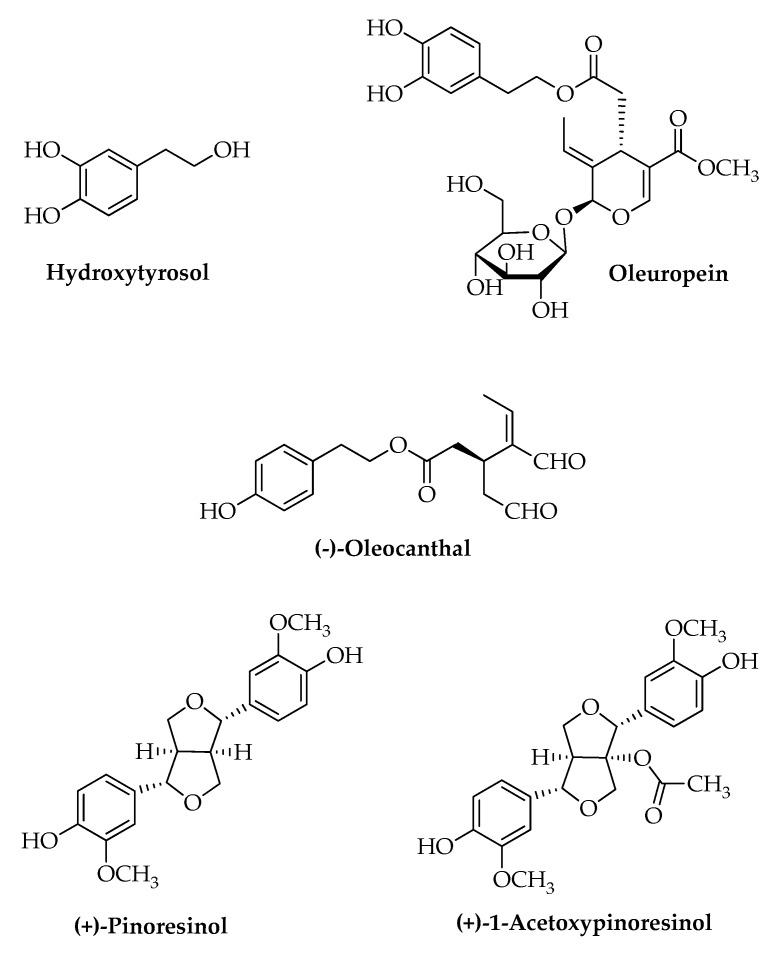
Chemical structure of the main phenolic compounds found in *Olea europaea* L.

Among the minor components, the phenolic ones are relevant for the health effects attributed to EVOO (Table 1) [1]. In particular, epidemiological studies indicate that dietary consumption of phenol enriched EVOO has a cardioprotective effect in Mediterranean populations. The minor polar compounds include different subclasses among these: secoiridoids that are the dialdehydic form of decarboxymethyl elenolic acid linked to *ortho*-diphenolic and/or phenolic alcohols, such as OLE aglycone and oleacein, deacetoxyoleuropein, oleocanthal (OLC) (tyrosol linked to elenolic acid), and traces of ligstroside aglycon (tyrosol linked to elenolic acid). Another class is represented by phenolic alcohols, with hydroxytyrosol (HT), and tyrosol (TYR), together with their secoiridoid precursors and traces of phenolic acids such as gallic acid, protocatechic acid, *p-*hydroxybenzoic acid, vanillic acid, caffeic acid, syringic acid, *p*- and *o*-coumaric acid, ferulic acid, and cinnamic acid. The flavonoids class is represented in traces; luteolin and apigenin are the flavones found in greater amounts. The last class is composed of lignans, and the most representative compounds in EVOO are acetoxypinoresinol and pinoresinol [3]. Among the minor polar compounds, HT and OLE are widely studied; in addition to the recently investigated OLC, they have been studied for their specific anti-inflammatory properties. In detail, HT and OLE (Figure 1) are valuable compounds for their high antioxidant capacity and for metal-chelating and free radical scavenging activities. HT is a molecule containing an *ortho*-diphenolic group that plays a significant role in EVOO. Its high antioxidant activity is due to its ability to scavenge reactive oxygen species (ROS) and stabilize oxygen radicals with an intramolecular hydrogen bond [4]. The above described lignans, pinoresinol and acetoxypinoresinol, also show antioxidant capacity [5].

**Figure 2:**
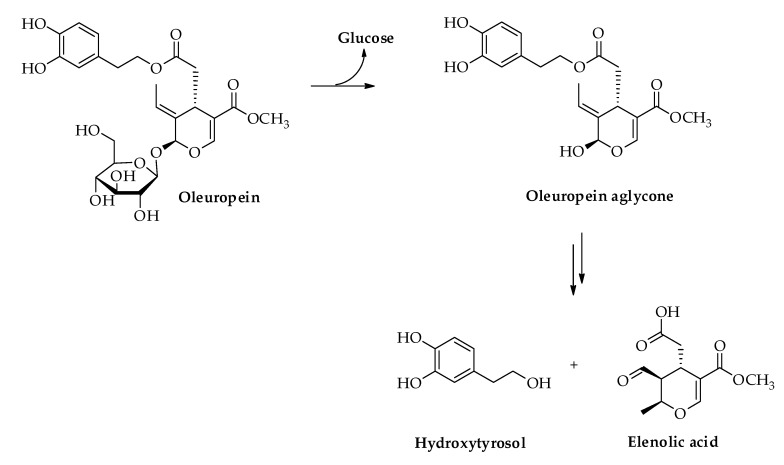
Enzimatic conversion of oleuropein into hydroxytyrosol.

Interestingly, during fruit maturation and olive oil production, the enzymatic systems present in the fruit are able to hydrolyze OLE first into its aglycone form, and then into HT together with glucose and elenolic acid, as depicted in Figure 2 [6]. Due to its hydrophilic character, HT is abundant in olive oil by-products and in particular in olive oil wastewaters, thus representing a precious source from which to extract this valuable compound [7].

This review is focused on the description of the beneficial effects of the main polyphenols found in EVOO, olive oil by-products and olive leaf, analyzing the beneficial properties of HT and OLE in detail.

The extraction procedures used to retrieve polyphenols from olive waste, leaf, and the fruit have recently been improved, and traditional systems have been optimized by newer technologies such as membrane separation techniques developed to fractionate olive mill wastewaters. The benefits achieved with the newer methods include lower energy consumption and no necessary additives or phase change [4,8].

**Figure 3:**
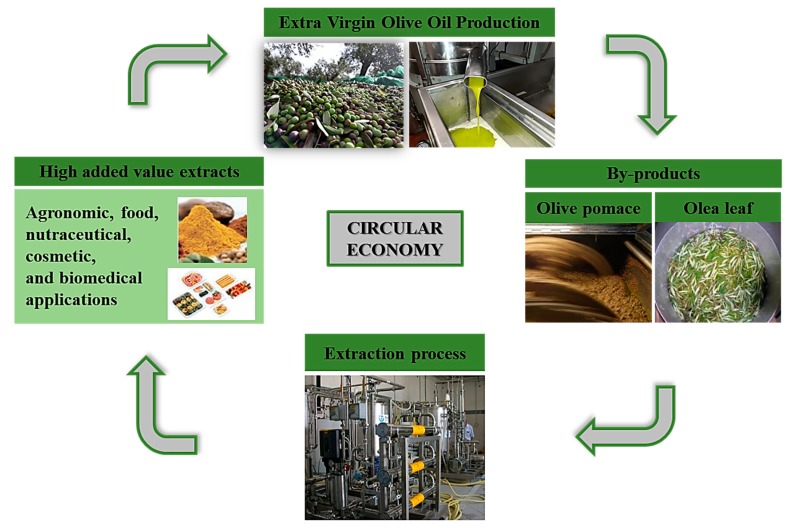
Circular economy platform based on green technologies, for the recovery of active molecules from olive leaf and processing by-products, useful in the food, nutraceutical, cosmetic, and biomedical fields.

This paper envisages the characterization and use of EVOO as a natural functional food, and the characterization and use of active extracts/ingredients obtained from olive leaf, pitted and de-oiled olive paste and juices, and from secondary matrixes (olive biphasic pomace and leaf), through sustainable green technologies. Fractions enriched in EVOO’s minor polar compounds, in OLE from leaf and in HT from de-oiled and pitted olive paste and the lipophilic derivatives, will be characterized and described from the chemical point of view, as quali-quantitative content in bioactive components and for their biological and nutraceutical activities (Figure 3).

The technical, economic, and environmental evaluation of the *Olea europaea* L. platform requires a material and energy flow analysis of the production cycle. The methodology used is the Membrane Filtration Absorption (MFA), which can evaluate the energy and material flows in a well identified system [9,10] that is the production process of the polyphenols from *Olea europaea* L., accounting for the inputs (consumption of material and energy resources consumption) and the outputs (waste products) of the process.

Data and methods concern the extraction of polyphenols from olive oil by-products based on new sustainable technologies with a water extraction and membrane separation system. Previously, scientific reports considered the same procedure for olive-oil waste-water polyphenol purification at a laboratory scale [11,12]. This innovative industrial process has instead been implemented at an industrial level by using physical technologies defined as BAT (Best Available Technology) and recognized by the EPA (Environmental Protection Agency) [13,14].

In the production process, the yield in quality EVOO, stands near 10–14%. Generally, the recovery process is based on membrane technologies applied to aqueous extracts obtained in a pneumatic extractor and then purified by filtration. The first phase of olive-oil production, from olives to oil and wet pomace, has only been considered in the material data (as Figure 2 shows) and it emerges that from the processed olives, by the use of a two-phases system, the wet-pomace can be extracted at the rate of 80% [15]. The remaining share represents the EVOO and olive oil (near 20%). Regarding the process, from wet olive pomace to the main output, the yield is very low and approximately 4.4%. The Olea by-products recovery process includes demineralized/pure water production and a final reverse osmosis (RO) phase. The chemical recovery and the production of energy should be a continuous process of interaction between green technology and environmental and economic sustainability, making this integrated platform highly innovative and consistent with the principles of the circular economy, with the development of new business activities. The results have highlighted that this platform can produce up to 6000 kg of standardized polyphenol fractions, useful in food and nutraceutical application; moreover, each residue of the process (water, olive cores, destoned pulp) comes into an innovative use in the same and/or other processes according to the circular economy models. The conclusion underlines the main positive features of this sustainable model, the eco-innovation of the process and the economic and environmental advantages consisting in reducing waste, water, and energy consumption. Operating plants that produce standardized natural fractions in HT and OLE contents, are in Spain and Italy and commercial fractions, in sharp increase in consumption are, for example, Phenolea Active, and OleaFit™ standardized in various bio-active compounds content.

## 2. Methods

Current literature analyzing the beneficial effects of the minor polar compounds of EVOO has been contextualized in this review. Specifically, the search was conducted using digital libraries such as Medline (*Pubmed*) and *Scopus*. The search examined studies published until May 2019, utilizing the words: *Olea europaea* L. minor polar compounds of extra-virgin olive oil, HT, OLE, OLC, lignans, biological activities of extra-virgin olive oil, and Olea by-products. Specific research related to the topic of this review carried out by all authors was also described.

## 3. Health Effects of Phenolic Compounds Found in Extra-Virgin Olive Oil (EVOO)

The cardioprotective properties of the Mediterranean diet (MD), related mostly to the beneficial effects of EVOO, were demonstrated for the first time in the Seven Country Study of Cardiovascular Disease (SCSCD) [16]. The MD consists of a balanced consumption of fruit, vegetables, legumes, and cereals, associated with a large assumption of bluefish and EVOO (the last as the main source of fats), reduced consumption of red meat and dairy products, and moderate intake of alcohol, mainly red wine. The MD has an important effect on maintaining health and increasing longevity, as cited by the United Nations Educational Scientific and Cultural Organization (UNESCO) in 2010 [17,18].

**Figure 4:**
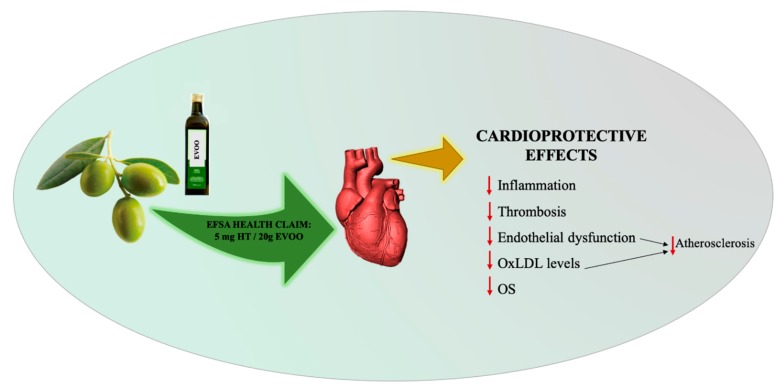
EVOO and its cardioprotective action on the CV system. EVOO, Extra-virgin olive oil; HT, hydroxytyrosol; OxLDL, oxidized LDL; OS, oxidative stress.

In the last few decades, numerous epidemiological studies [19] and meta-analyses [20], as well as intervention trials [21,22,23], confirmed this observation, pointing out the protective role of the MD on primary [24] and secondary [25] prevention of CVDs (Figure 4).

The PREDIMED study investigated, on 7477 subjects at high risk of CVDs, the protective effect of the MD supplemented with EVOO or nuts on major cardiovascular (CV) events (such as stroke, myocardial infarction, or death from CV causes). The authors highlighted that the incidence of major CV events was significantly decreased in subjects following the MD supplemented with EVOO or nuts compared to those following a reduced-fat diet, confirming the beneficial effects of the MD for primary CV prevention [24].

A prospective randomized trial [25] compared a MD enriched with alpha-linoleic acid to a prudent diet, in regards to their respective effects on secondary prevention after myocardial infarction, concluding that the alpha-linoleic acid-enriched MD was more effective in secondary prevention of acute coronary events and death, since it has been hypothesized that linoleic acid reduces the incidence of fatal arrhythmias [26].

In our study, we observed for the first time the health effects of the Italian Mediterranean diet (IMD) and the Italian Mediterranean organic diet (IMOD) in healthy subjects and in stage II–III chronic kidney disease (CKD) patients, staged according to the Kidney-Disease Outcomes Quality Initiative (K-DOQI) guidelines [27]. Specifically, we demonstrated a significant reduction of phosphorus, total homocysteine (Hcy), and albuminuria, as well as an improvement of body composition (a significant increase in lean mass percentage and a decrease in fat mass both in kg and in percentage) after two weeks of IMOD treatment. The improvement of all these clinical parameters is associated with lower CV risk, highlighting the role of the IMOD in the prevention of CVDs [22]. The IMOD would seem to induce a slowing down of CKD progression.

In our study, we subsequently confirmed that the IMOD in CKD patients on conservative therapy represents a useful tool for the prevention of CVDs, inducing a significant reduction of serum Hcy influenced by the methylenetetrahydrofolate reductase (MTHFR) genotype [23]. Hcy causes endothelial dysfunction through production of ROS, which occurs during the autoxidation process, accelerating atherosclerosis [28,29].

In 2011, the European Food Safety Authority approved some claims reported by the Commission Regulation no. 432/2012 concerning the benefits of bioactive compounds found in foods including EVOO phenols and, in particular, of HT and OLE, supporting their relevant role for human health [30]. The health effects are protection of LDL from oxidative damage, maintenance of normal blood HDL cholesterol concentrations, maintenance of normal blood pressure, anti-inflammatory properties, contribution to upper respiratory tract health, maintenance of normal gastrointestinal tract function, and contribution to body defenses against external agents. These beneficial effects are obtained with a daily intake of 20 g of EVOO, containing 5 mg of HT and its derivatives [31]. Indeed, the oxidized LDLs (OxLDLs) binding with the receptor LOX-1 (receptor OxLDL-lectin-similar to receptor-1), stimulate endothelial expression and secretion of pro-atherogenic enzymes. This bond induces the production of superoxide and the reduction in local nitric oxide (NO) concentration. LOX-1, which causes a rapid rise in ROS levels through membrane-bound NADPH oxidase (NOX), is a specific endothelial scavenger receptor involved in the initial process of atherosclerotic plaque formation [32].

EVOO extracts were tested in vitro on human endothelial cells (HUVEC) to evaluate their antioxidant capacity [33] and their ability to modulate the cellular expression of ICAM-1 and VCAM-1 in a pro-inflammatory environment, in order to investigate their anti-atherogenic effects [34]. In order to better understand the differences among the in vitro and in vivo effects of EVOO, it will be necessary to compare various monocultivar in phenolic content.

Despite the biological activities of dietary phenolics, only a few studies have been carried out to investigate their absorption in humans after ingestion. A specific study on olive oil phenolics was performed by Visioli et al. [35], demonstrating that they were absorbed in humans and excreted in the urine as glucuronide.

As far as the biological activities of olive oil are concerned, a review by Covas et al. [36] examined 15 human studies and the majority indicate that olive oil (rich in phenols) is superior to seed oils and olive oil with low-phenol content. This superiority was attributed to the reduction of CV risk factors, such as reduced plasma LDL, improved endothelial function and a decreased prothrombotic environment.

Polyphenolic metabolites of EVOO after ingestion were methylated, sulfonated, or glucorinated; in vitro studies [37,38] have shown that these metabolic modifications do not inhibit their biological activities in humans. Moreover, the parent compounds and the metabolites derived from EVOO are capable of reaching a concentration at tissue level (mainly in the gastrointestinal and CV systems) able to exert antioxidant and anti-inflammatory actions, by modulating intracellular signaling [39].

Pharmacological studies concerning the activities of phenolic compounds are increasing, since they seem to have potential cardioprotective and chemopreventive actions.

HT and OLE are able to inhibit copper-induced LDL oxidation at low concentrations and show a powerful ability to chelate metals and scavenge free radicals [26,27]. In particular, a specific study carried out by Franconi et al. [40] demonstrated that the concentration capable of reducing copper-induced LDL is similar to that measured in human plasma after EVOO intake [40,41].

A study on 10 healthy postmenopausal women compared the effects of high-phenol EVOO (592 mg total phenols/kg) with a low-phenol EVOO (147 mg total phenols/kg). Daily dose of EVOO was 50 g per day for a period of 8 weeks. Oxidative DNA damage was evaluated by monitoring peripheral blood lymphocytes. Subjects who took high-phenol EVOO had oxidative DNA damage reduced by 30% compared to subjects treated with low-phenol EVOO. This study demonstrated a protective role of EVOO phenols on oxidative damage in healthy postmenopausal women. Moreover, subjects who consumed high-phenol EVOO had a significantly increased urinary excretion of HT compared to low-phenol EVOO [42].

These data were confirmed in a randomized trial in 200 healthy male subjects that evaluated the effects of olive oil phenol content on lipid oxidative damage and plasma lipid levels. The enrolled subjects were randomized into three groups based on the content of phenols in olive oil and took 25 mL of oil per day for a total of 3 weeks. The three types of olive oil were the following: low-phenol olive oil (2.7 mg/kg of olive oil), medium-phenol olive oil (164 mg/kg of olive oil), and high-phenol olive oil (666 mg/kg of olive oil). The authors highlighted that the biomarkers of oxidative stress were reduced proportionally to the phenol content, while the HDL-cholesterol levels increased directly to the phenol content of the olive oil. Triglycerides were reduced in all the three groups examined. Therefore, this study confirmed that phenolic content improves lipid oxidative damage and lipid profile [43]. Several studies showed that a constant consumption of EVOO is associated with a reduction in the molecules involved in inflammatory processes related to atherosclerosis, by the downregulation of NF-kB [44,45].

The close correlation between inflammation, endothelial dysfunction, and CVDs is well known [46,47]. In the study conducted by Brunelleschi et al. [48], phenolic-rich EVOO inhibits, in a concentration-dependent manner, the nuclear translocation of the p50 and p65 subunits of the NF-κB complex within monocytes and monocyte-derived macrophages (MDM) of healthy subjects. This inhibitory effect was especially evident when the cells were stimulated by phorbol-myristate acetate (PMA) and obtained at EVOO extract concentrations similar to those measured in human plasma after a daily ingestion of EVOO. This inhibitory action is comparable to the effect exerted by ciglitazone, a PPAR-γ ligand.

Moreover, OLE, the most abundant polyphenol in EVOO, has shown to significantly increase lipopolysaccharide (LPS)-induced NO production, a bactericidal and cytostatic agent whose heightened expression increases macrophages’ functional activity [49].

Western blot analysis of cell homogenates, and coincubation of bacterial LPS challenged cells with L-nitromethylarginine methylester (a nitric oxide synthase, iNOS inhibitor), showed how OLE directly stimulates the inducible form of the iNOS enzyme, leading to the above-mentioned enhancement of macrophage function [49].

The incidence of chronic illnesses related to aging and unhealthy lifestyles is on the rise, but increasing data are showing how the intake of secoiridoid-rich EVOO may help to prevent or even treat chronic non-communicable diseases (NCDs), in which the inflammatory component is directly involved in their onset and progression [50,51].

Another secoiridoid of considerable interest is OLC because recent studies have highlighted its pharmacological properties and its mechanisms of action, showing the preventive effect on inflammation, oxidative stress, specific types of cancer, neurodegenerative and rheumatic diseases. OLC is one of the components of EVOO and it is responsible for the pungent character of this food. This perception seems to be due to the presence of a specific OLC receptor present in the oropharyngeal region [52]. This receptor seems to be the transient receptor potential channel, subfamily A, member 1 (TRPA1) [53]. It is hypothesized that the different sensitivity in the perception of the pungent taste of OLC may be related to the inter-individual variations in the expression of the TRPA1 receptor in the oropharynx [53]. This sensation is similar to that observed after taking ibuprofen (non-steroidal anti-inflammatory drug), and from this observation, some authors have hypothesized that the two substances could have the same biological activity [54]. Despite the structural differences, both molecules inhibit the same cyclooxygenase enzymes involved in the biosynthesis of prostaglandins. In detail, both OLC enantiomers induce the inhibition of cyclooxygenase (COX) 1 and 2, but in vitro have no effect on lipoxygenase. The inhibition of COX 1 and 2 is dose-dependent: an OLC concentration equal to 25 μM inhibits COX activity by 41% and 57% vs. 25 μM of ibuprofen which inhibits COX by 13% to 18%, respectively [55].

Therefore, the authors have hypothesized that the long-term consumption of OLC may protect against the onset of certain pathological conditions, due to its biological action similar to ibuprofen [56,57]. The dosage that seems to be active is 9 mg per day, which corresponds to 10% of the dose of ibuprofen taken by adults to counteract pain. In the literature, it is known that a constant low dose of aspirin (another COX -inhibitor) induces CV protection [54]. Therefore, it is hypothesized that a long-term OLC consumption may also exert a cardioprotective action.

To date, the OLC-induced cardioprotective action has been little investigated. In fact, a single study highlights the possible protective effects of OLC in atherosclerotic CV disease [58]. This disease is a chronic inflammatory process that affects the vessel walls and begins with damage to the endothelium. Endothelial damage mainly involves platelets [58,59]. Recently, Agrawal K. et al. [60] have shown, in a randomized clinical trial, that the intake of 40 mL per week of EVOO rich in OLC can influence platelet aggregation responses in healthy male adults, confirming previous data obtained from animal studies [61]. Currently, the analytical methods for the OLC assay are not standardized and, in literature, not all the studies reporting the content of EVOO minor polar compounds show this data. Therefore, the value of 9 mg per day is probable but not yet defined. The concentration of OLC is usually low in fresh EVOO and increases during EVOO storage due to hydrolysis of secoiridoids that enhance HT [39].

A study carried out by Carrrasco-Pancorbo et al. [62] has demonstrated the antioxidant activity of pinoresinol and acetoxypinoresinol using the DPPH (2,2-diphenyl-1-picrylhydrazyl) method and evidenced that the absence of the acetyl group in pinoresinol is relevant for its activity [5,62]. Further studies demonstrated that pinoresinol also shows in vitro anti-inflammatory activity [63]. In vitro studies show that both acetoxypinoresinol and pinoresinol have chemopreventive activity in breast cancer by decreasing the levels of fatty acids synthase in the HER2 gene that is over-expressed in breast cancer cells [64]. A study conducted in 2008 [65] demonstrated the capacity of pinoresinol, in synergy with other phenolic compounds present in olive oil extracts, in decreasing proliferation and inducing apoptosis of human colon cancer cells.

A limited number of available randomized controlled trials (RCTs), show EVOO’s action in secondary prevention of diseases related to atherosclerosis and there are no RCTs aimed at assessing the minimum daily EVOO intake required in order to have an anti-inflammatory and cardioprotective action [66].

Evidence indicates that regular consumption of EVOO is associated with a reduced risk of developing NCDs. In the field of NCDs, disorders like cancer, CKD, arterial hypertension, and metabolic syndrome deserve a special mention [67].

The European Prospective Investigation into Cancer and Nutrition (EPIC) study has indicated the possible correlation between cancer and nutrition, examining lifestyle, nutritional status, type of diet, medical history, anthropometric parameters, and biological samples. This study was conducted on 521,000 subjects (one of the largest cohort studies in the world) enrolled from 23 centers in 10 western European countries. Following enrollment, the participants were contacted at regular intervals every 3–5 years (depending on the country or center) to obtain information on various aspects of their lifestyle, which may have changed over time. To date, the data collected indicates that the MD is the most effective food model in cancer prevention. There is also evidence that consumption of flavonoids reduces the risk of gastric cancer [68,69].

The EPIC study Italian cohort, composed of 47.749 volunteers, was evaluated for eating habits and lifestyle. The authors concluded that the food pattern “Olive oil and Salad” food pattern, mainly based on the consumption of raw vegetables, EVOO and legumes is associated with lower mortality in the elderly and a lower risk of developing colorectal cancer. The latter has a lower incidence in regular yogurt consumers for the probable protective action exerted by probiotics against NCDs [70,71].

These data were confirmed in a cohort of 5,611 Italian elderly subjects (aged ≥ 60 years), where a Cox model showed that a high consumption of olive oil, fresh vegetables, soup, and poultry was inversely correlated with mortality from all causes. On the other hand, the food pattern “Pasta and Meat” characterized by a high content of pasta, tomato sauce, red and processed meat, added animal fats, white bread, and wine was associated with an increase in mortality from all causes. The authors recommend a high consumption of olive oil, fresh vegetables, and poultry in the geriatric population due to its effects on health [72].

In the EPICOR (long-term follow-up of antithrombotic management patterns in acute coronary syndrome patients) study, the authors enrolled 29,689 Italian women from northern, central, and southern cities, evaluating the possible associations between assumption of EVOO, vegetables and fruit, and incidents of coronary heart disease (CHD). The mean follow-up period was 7.85 years. They demonstrated that women who consumed vegetables and olive oil in the highest quartile had a reduced risk of developing CHD. This study confirms the protective effect for CVDs in primary and secondary prevention, related to the consumption of vegetables and olive oil [73].

Our project called EXTRANUTRAOILS is evaluating the impact of EVOO with health claims (natural functional food) in CKD patients, to investigate the effects of high-phenol EVOO on the progression of CKD and its complications (Table 2).

### Impact of Olive Oil and Its Derivatives on Gut Microbiota Composition

One of the most densely populated human ecosystems is the gastrointestinal tract. It boasts the presence of about 10^13^ microbial species within it, and is called the “gut microbiota” [74].

The study of the link between human gut microbiota and health status has attracted considerable interest in the scientific community over the past 15 years. It is essential to understand how different nutrients impact the gut microbiota composition which in turn influences the onset and progression of chronic non-communicable diseases [75]. Currently, the knowledge on the gut microbiota indicates that it can interact with the host both directly and indirectly. In fact, it is able to release bioactive molecules that modulate numerous biological responses, involving several systems and functions such as the immune system and/or energy homeostasis [76]. Some nutrients can influence the composition of gut microbiota; among these, we find olive oil and its derivatives [77].

Pallara G. et al. [78] evaluated the polyunsatured fatty acid (FA) profile derived from ruminant livestock, after the administration of feed supplemented with stoned olive pomace (SOP), which represents a waste deriving from the processes of conversion from olive or olive oil. They concluded that feeds supplemented with SOP decreased the production of unsaturated FA in a dose-dependent manner through the modification of gut microbiota composition. Therefore, functional lipids can be produced from meat and dairy products through animal feed supplementation.

N. Martinez et al. [79] performed an animal study to compare the effects of standard diets versus high fat diets (enriched with EVOO, refined olive oil or butter) diets, on gut microbiota composition. In order to evaluate the possible variations on gut microbiota caused by different diets, they sequenced mice fecal 16S rRNA. The group fed with a high fat-diet enriched with refined olive oil, showed significantly higher levels of total cholesterol compared to the EVOO diet group. Moreover, the high-fat diet enriched with refined oil group showed a greater presence of Desulfovibrionaceae, Spiroplasmataceae, and Helicobacteraceae families. The authors showed a direct relation between the quality of fats in the diet (in this case refined olive oil and EVOO), some laboratory parameters, and the presence of certain taxa. For this reason, it becomes increasingly clear that the minor polar compounds present in EVOO are able to positively modulate the gut microbiota.

Prieto et al. [80] investigated, in Swiss Webster mice, the effects (on hormonal, physiological, and metabolic parameters) of a diet having EVOO as the main source of fat, compared to a diet enriched with butter. Moreover, the authors analyzed fecal DNA e 16S rRNA genes. In the mice with butter diets, there were higher values of systolic blood pressure and a greater percentage of Desulfovibrio sequences compared to the mice on EVOO diet. In addition, in mice with EVOO diet, the authors observed reduced plasma levels of insulin and leptin. The concentration of leptin was inversely related to *Sutterellaceae, Marispirillum*, and *Mucilaginibacter dageonensis*. Therefore, the intake of EVOO would seem to influence the composition of the intestinal microbiota, which in turn can positively affect health status.

In spontaneous hypertension rats, M. Hidalgo et al. [81] investigated the effects of EVOO on gut microbiota composition and blood pressure levels. After 12 weeks, the rats fed with the diet enriched with EVOO had significant difference in *Lactobacillus* and *Clostridia XIV* percentage with respect to rats fed with a standard diet. Moreover, the abundance of *Clostridia XIV* was inversely related to systolic blood pressure values.

In a randomized, double blind cross-over human study [82] conducted on 10 hypercholesterolemic subjects, the authors evaluated the effects of the consumption of 25 mL/day of olive oil for three weeks on human intestinal immune function. The authors studied the effects of three olive oils (OO) differing in their content of phenolic compounds: OO varieties containing 80 mg phenolic compounds/kg, OO containing 500 mg phenolic compounds/kg from OO and OO containing a mixture of 500 mg PC/kg from OO and thyme. The authors concluded that the consumption of olive oil with a high content of phenolic compounds induces an increased stimulation of the intestinal immune system.

## 4. Health Effects of Phenolic Compounds Present in Olea By-Products and Waste

Olea by-products and waste are precious sources of bioactive compounds that could be selectively recovered and reused for industrial applications. These principles are the pillars of the circular economy, a model of economy where by-products are not waste but resources to be valorized and reused (Figure 3).

The material balance of the process is equal to a total of 73,200 kg as input and output, of which 2,940 kg are the main outputs. It should be noted how this multifunctional platform is highly innovative and in line with circular economy principles. Each residue of the process (water, olive stones, destoned pulp) would come into new use in the same and/or external processes, according to the “zero waste” model. Indeed, after the extraction of the bioactive fractions, residues of the olive oil mill can be used as animal feed, compost, or other agricultural or agro-industrial products and/or be exploited as energy sources in the same biorefinery or sold for other economic activities.

Olive tree cultivation is particularly widespread in the Mediterranean Basin and provides a strong contribution as a source of polyphenols. After the production of olive oil, olive pulp and olive oil wastewaters are obtained as by-products in large quantities, representing a great environmental problem in consideration of their high toxicity [83]. Olive oil by-products such as olive mill and leaves are allowed for production of feed, cosmetics, food, and nutraceuticals, whereas waste and olive oil waste waters are only allowed for use in agronomy. The importance and use of waste from the production of oil, as a resource and source of polyphenols, has been described by Cecchi et al. in a recent study [84], which showed that only 0.5% of the total polyphenols present in olives is found in the extracted oil, while the remainder is waste that can be used for the formulation of supplements for the nutraceutical sector.

Regarding the recovery of waste from oil production, there are studies in literature concerning the treatment of wastewater. A recent study demonstrated that the retentates obtained after microfiltration, ultrafiltration, nanofiltration, and reverse osmosis are stable; HT recovered was stable for 24 months and the process showed a good reproducibility [85].

Among the wastes from oil production, in addition to olive mill wastewaters, there is patè, a particular dried olive pomace containing high amounts of HT and OLE. It has been estimated that 1.0 g of patè contains the same amount of polyphenols as 200 g of EVOO, thus representing a good source to be used for industrial applications [86]. A recent study indicated that extracts obtained from olive mill wastewater exhibited cytoprotective effects in PC12 cells [87]. Similarly, they showed antibacterial activity [88].

**Figure 5:**
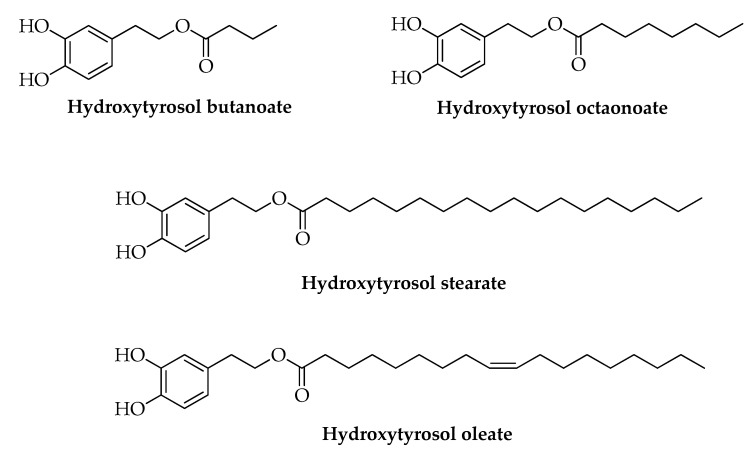
Lipophilic HT derivatives.

HT is a small phenol that can be recovered from olive oil by-products and used as starting materials for the preparation of novel bioactive compounds [89,90,91,92]. Thanks to the presence of the alcoholic group, it is possible to prepare the corresponding alkyl derivatives showing better lipophilicity and bioavailability than HT. Figure 5 describes some examples of saturated and unsaturated lipophilic HT derivatives. These compounds could be obtained by a selective esterification of HT with acyl chlorides of different chain length in order to modulate their lipophilicity [89,93]. Interestingly, the same functionalization has been introduced in *Olea europaea* L. extracts enriched in HT recovered by olive oil by-products, and their antiproliferative activity on the human colon cancer cell line HCT8-β8 was evaluated [7]. The experimental results indicated that both the presence and the length of the alkyl chain exert a relevant role on the antiproliferative activity [94]. In a similar manner, HT stearate and HT oleate showed anti-inflammatory activity [95]. To date, it appears that experiments on animal and human models are lacking (Table 3) [96].

## 5. Health Effects of Phenolic Compounds Present in Olea Leaf and Olea Leaf Extracts

Olive leaves, deriving both from the processing of olives and from pruning practices, can be definitely considered a waste of the olive supply chain. These vegetal tissues are a valuable source of bioactive compounds including phenols with low molecular weight [97]. In olive leaf, the prominent constituent is the secoiridoid OLE, which by enzymatic or chemical hydrolysis produces the aglyconic form present in the oil, HT, elenolic acid, and glucose [6,49].

In a 2016 study, several extracts from olive leaf were analyzed. Qualitative differences in total polyphenols and in OLE content were found. In particular, polyphenols varied from 7.87 to 34.21 mg/g, while OLE varied from 2.79 to 21.03 mg/g depending on the kind of leaf (fresh, refrigerated, dried, frozen, or lyophilized), cultivar, sampling time, and production area. This study has pointed out that OLE and stability are related to the extraction temperature and drying process [7].

Many benefits are related to the properties and chemical characteristics of olive leaf. For this reason, many studies are focused on the use of olive leaf for human consumption. In the last few years, olive leaf extracts have been used by the food industry as foodstuffs or food additives [98] producing functional foods with health properties. In a 2017 review, all the studies carried out on the benefits of olive leaves and their extracts were collected [99]. To date, olive leaf extracts have been sold as dried leaves, powders, extracts, or tablets used as herbal teas or food supplements, available all over the world. Extracts of hot fresh water leaves are eaten to increase diuresis and treat hypertension and bronchial asthma [99]. Olive leaves also affect metabolism, so they have been used as a traditional herbal medicine for years. Many studies, both in vitro and in vivo, demonstrated their important biological properties, including radio-protective, anti-proliferative, and cytotoxic effects on cancer cells; anti-fungal activity; and anti-atherosclerotic, hypoglycemic, and cardioprotective effects [99]. As reported above, the olive leaves antioxidant activity is ascribed to secondary metabolites, in particular OLE and its ability to chelate Cu and Fe metal ions, which catalyze free radical generation reactions [100]. Janahmadi et al. [101] showed that OLE intake in rats with a permanent ligation of left main coronary attenuated heart failure progression through antioxidative and anti-inflammatory effects. In alloxan-diabetic rats, the intake of olive leaf extract in a concentration of 16 mg/kg body weight influences their lipid profile, improving hypercholesterolemia associated with hyperglycemia [102]. A recent study reported that 20 healthy subjects after an intake of 20 mg of OLE before lunch showed an improved post prandial glycemic profile by reducing glucose and increasing insulin and GPL-1 [103]. In overweight middle-aged men at risk of developing diabetes, supplementation with olive leaf polyphenols for 12 weeks significantly improved insulin sensitivity and pancreatic β-cell secretory capacity [104].

What is of translational importance is that OLE was found to be a powerful sensitizer of doxorubicin (DXR)-mediated killing of prostate and breast cancer cells. In fact, in a first study, 200 μg/mL of OLE, was able to inhibit cell proliferation at very low doses of DXR (3–12.5 nM). In a second study in a breast tumor xenograft in mice, the intraperitoneal injection of the combination of 1.5 mg/kg of, followed by 50 mg/kg of OLE, decreased the volume of the tumor threefold [105,106]. A recent investigation demonstrated that olive-leaf ingredients, such as HT, are good antioxidants for food lipids even at very low doses (<100 mg kg^−1^), without cytotoxic effects, nor do they inhibit probiotic lactating producing bacteria.

Furthermore, olive leaf-derived phenolic compounds have shown significant antimicrobial properties, thus playing an important role in the control of food processing and preservation during storage, as well as in counteracting pathological microorganisms like *Helicobacter pylori* and other food-borne pathogens [107]. As a consequence of their recognized nutraceutical activities, olive-leaf extracts containing polyphenols can be used as foodstuff ingredients. OLE, and in particular its aglycone obtained by enzymatic hydrolysis of pure extracts from olive leaves, as previously reported [108], also showed an anti-amyloid effect, resulting in protection against the cytotoxic effects of amyloid aggregates [109,110], as well as having an autophagy inducer effect by modulating the AMPK/mTOR pathway and by activating autophagy gene expression mediated through sirtuins or EB transcription factor [108,111,112]. These last actions suggest that OLE aglycone may have a neuroprotective action in diseases such as Alzheimer’s, characterized by amyloid deposition and autophagy impairment, contributing to a decrease in aggregated protein and to a reduction in cognitive impairment in in vivo models [113]. Another interesting effect of OLE aglycone is its modulation in the tumor microenvironment to control tumor angiogenesis as reported by Margheri et al. in 2019 [114]. They showed that the treatment with OLE aglycone of “senescence-associated-secretory-phenotype” (SASP) fibroblasts, an accepted cellular senescence model decreased the release of SASP pro-angiogenic factors in cell media, inhibited dependent cell invasion, and inhibited formation of capillary-like structures of endothelial cells exposed to the same media, suggesting a mechanistic interpretation of the anti-angiogenic activities for cancer prevention by olive oil polyphenols.

Moreover, as recently reported in a review that summarized the existing in vitro and in vivo studies [115] on OLE and its metabolite HT [116], these compounds not only exert a chemopreventive action “per se” (by increasing apoptosis and decreasing proliferation and viability), but they may also be used as adjuvants to conventional antitumoral therapies [117,118]. OLE, at non-toxic doses, blocks the AKT pathway and may act synergistically with several current chemotherapies used against BRAF melanoma cells, allowing a decrease in drug dosage that can lower the adverse effects on non-target cells and reverse resistance towards conventional agents such as 42-O-(2-hydroxyethyl) rapamycin (RAD001) and dacarbazine (DTIC).

In the context of anticancer therapy, the anthracycline DXR has a limited use as a chemotherapeutic agent due to its cardio-toxic effects. Consequently, sustained research has been focused on identifying effective drugs and strategies in order to reduce DXR toxicity without compromising its antitumor efficacy. Some studies reported that OLE prevented cardiomyopathy caused by chronic DXR toxicity and, by attenuating inflammatory development and the degenerative myocardial lesions, preserved left ventricle contractility and/or [119] acted as a sensitizer of DXD-induced death of cancer cells.

In addition, [118] reported that leaf extract enriched in OLE was even more effective than OLE alone against melanoma cells, probably because of the co-presence of other polyphenols, suggesting that the combination of several components (rather than any single one alone) might be the ultimate chemopreventive agent (Table 4).

## 6. Bioaccessibility and Bioavailability of Olea Minor Compounds

Among the minor polar compounds of Olea products HT, Tyr, and OLE have different bioaccessibility and bioavailability. One of the first studies on the bioavailability of the minor polar compounds was conducted by Visioli et al. from 2000, which showed that HT and Tyr are dose-dependently absorbed [35]. In a recent review, studies on the bioavailability of principal Olea minor compounds—such as HT, Tyr, and OLE—in olive oil were collected [120]. In a 2016 study, the plasma levels of HT were evaluated after ingestion of olive oil and EVOO. The pharmacokinetic results show that it was not possible to detect HT in the blood after the ingestion of ordinary olive oil, unlike the results obtained after the ingestion of EVOO [121].

As many authors reported, the bioaccessibility regards the amount of component(s) of interest that is (are) released from the food matrix into the gastrointestinal (GI) tract, whereas the bioavailability is the quantity of the same digested compound(s) that is(are) absorbed and metabolized within the human body. As for OLE, some authors reported that, under in vitro gastrointestinal conditions, it was the most resistant compound among those present in olive leaves, varying its content between 26% and 61%, depending on the type of extract during the first hour [122]. The same authors found that the intestinal phase affected OLE more than the gastric one probably due to pancreatic enzymatic activity and alkaline pH contributing to a reduction of its bioaccessibility, so that at the end of the digestion processes only 10% of OLE was present.

HT and OLE bioaccessibility has been investigated in vitro by Jilani H et al. [123] in relationship to the potential use of *Saccharomyces cerevisiae* as a new carrier of OLE as means to protect their lasting antioxidant capacity during simulated gastrointestinal digestion.

Using an in vivo digestion condition, de Bock et al. [124] found that after ingestion of different doses of OLE and HT in capsule or liquid form, the plasma of subjects receiving liquid doses (2.74 ng/mL) showed detectable peak concentrations that were higher compared to those who consumed capsules (0.47 ng/mL). In fact, previous results showed the influence of the dose and formulation taken by the subjects, in addition to gender, in plasma concentrations. In the same study, the authors also noticed that the primary metabolites identified in urine and plasma were the conjugated metabolites of HT, mainly consisting of HT sulphated and glucuronidated compounds [125]. The gut microbiota is another factor that may influence the bioavailability and bioaccessibility of olive phenolic compounds by performing biotransformation to other active metabolites with interesting beneficial health properties in bowel diseases [126]. Using human fecal microbiota, an in vitro model [127] observed that OLE was rapidly deglycosylated until 6 h of incubation producing oleuropein OLE aglycone, that, on the other hand, was degradated into elenolic acid and HT by microbial esterase activity, until it disappeared after 48 h. On the contrary, HT, the main metabolite of OLE ester hydrolysis constantly increased during the same fermentation period. The same authors combined the in vitro colon fermentation studies with an in vivo intervention and found, after 3 weeks’ intake of phenol-rich olive oil, a significant increase in the concentration of free HT in the feces of the all participants in the study, confirming the in vitro findings.

Interestingly, Santos et al. [128] showed that conversion of OLE into HT was performed by lactic acid bacteria, in particular by *Lactobacillus plantarum*, and on the basis of this evidence, some authors recently developed oral granules for co-delivery of *L. plantarum* and a standardized olive leaf extract (Phenolea^®^Active F) in order to foster OLE metabolism and provide high levels of HT [129].

## 7. Conclusions

*Olea europaea* L. fruits and leaves are a matrix rich in bioactive compounds such as unsaturated acids, phenolics, phytosterols, tocopherols, and squalene. The main components are fatty acids, in particular oleic and linoleic acids, while secoiridoids, polyphenols, phenols, and lignans turned out to be minor polar compounds. HT and OLE are the most active compounds; HT is present mainly in fruits, olive oil, and olive oil by-products, while OLE is present in Olea leaves. Despite their low concentration, they are responsible for numerous health effects in humans. The chemical characteristics, functional or medical food properties, biological and biomedical activities of these compounds were described in this review with the aim to demonstrate how the olive tree can be a food species of great scientific and health interest.

EVOO is a functional food with legal health claims, certified as cardioprotective. These characteristics are related to the content of minor polar compounds, in particular to HT and its various derivatives. Therefore, among all dietary plans, a MD based on the daily consumption of EVOO as a source of fat, is an ideal dietary model for its beneficial cardioprotective effects, longevity, and prevention of NCDs.

Recent circular economy models promoting green technologies for the recovery of active compounds from by-products and waste are already operational in the olive-oil industry. Biological and biomedical activities of many secondary metabolites from *Olea europaea* L. have been scientifically demonstrated. Studies on the innovative use of standardized fractions in HT content as precursors for the synthesis of new biologically active molecules with rich bioavailability have already been processed. For this reason, the olive tree is an unmatched sustained resource for unique bioactive compounds with diverse health benefits.

## Figures and Tables

**Table 1:** Subclasses of EVOO minor polar components

EVOO Minor Polar Components	
**Secoiridoids**	(a) Oleuropein aglycone
	(b) Deacetoxy oleuropein
	(c) Oleocanthal and oleacin
	(d) Ligstroside aglycone
**Phenolics**	(a) Hydroxytyrosol
	(b) Tyrosol
	(c) Hydroxytyrosol glycole
**Phenolic acids**	(a) Gallic acid
	(b) Protocatechuic acid
	(c) *p*-Hydroxybenzoic acid
	(d) Vanillic acid
	(e) Caffeic acid
	(f) Syringic acid
	(g) *p*- and *o*-coumaric acid
	(h) Ferulic acid
	(i) Cinnamic acid
**Flavonoids**	(a) Luteolin
	(b) Apigenin
**Lignans**	(a) (+) Pinoresinol
	(b) (+) Acetoxypinoresinol

**Table 2:** Studies on extra virgin olive oil.

Extra Virgin Olive Oil					
Type of Study	Reference	Year	Type of Intervention	Primary Outcome	*p*-Value for Primary Endpoint
In vitro cell models	Manna, C. [41]	2002	Evaluation of effects of phenolic fraction extract from EVOO on oxidative damage in human erythrocytes and Caco-2 cells	Protective effects of EVOO phenolic fractions	Linear relationship between antioxidant capacity of EVOO phenolic fraction and *o*-phenolic content. *R*^2^ = 0.999
	Beauchamp, G.K. [54]	2005	Evaluation of effects of oleocanthal as modulator of inflammation and analgesia	Oleocanthal caused dose-dependent inhibition of COX-1 and COX-2 activities.	- N.A.
	Carrasco- Pancorbo, A. [62]	2005	Electrochemical study on the resistance of oxidative deterioration of VOO correlated to the presence of phenolic compounds	Ability of compounds isolated from VOO by measuring the radical scavenging effect on 1,1-diphenyl-2-picrylhydrazyl radical	- N.A.
	Vuorela, S. [63]	2005	Phenolic extracts isolated from bioactive sources have been studied for their antioxidant, antimicrobial, anti-inflammatory, and antimutagenic properties.	Phenolic extracts from oils, induced a decrease of proinflammatory mediators (prostaglandin E2). All tested extracts were safe. In fact, they did not stimulate mutagenic nor toxic action on Caco-2 cells or macrophages.	- N.A.
	Dell’Agli, M. [34]	2006	Evaluation of HT and OleA form EVOO in HUVEC	Expression of - ICAM-1 VCAM-1 at concentration (IC_50_ < 1 micro M) - -HVA - -E-selectin cell surface expression	- Downregulation of adhesion molecules associated with early atherosclerosis
	Franconi, F. [40]	2006	Whole virgin olive extracts studied to determine whether they maintain the antioxidant activity and whether this last is linked to MPC composition of a single virgin oil	Evaluation of oils derived from Taggiasca and Seggianese olive on human LDL	- In both tests, the oil extracts dose-dependently reduced malondialdehyde and conjugated diene generation - Seggianese extract was more active with respect to Taggiasca extract.
	Brunelleschi, S. [48]	2007	Evaluation of EVOO extracts rich in minor polar compounds (MPC-OOE) on human cells	NF-kB translocation in monocytes and monocyte-derived macrophages sampled from healthy subjects	- MPC-OOE extracts inhibits NF-kB translocation in human monocytes and MPC does not affect PPAR-γ in human monocytes and MDM p < 0.001
	Menendez, J.A. [64]	2008	Evaluation of EVOO phenolic effects on the expression of FASN in human breast cancer epithelial cell lines.	EVOO phenols: lignans, flavonoids, and secoiridoids suppress FASN protein expression in HER2 gene amplificated SKBR3 breast cancer cells	- Extracts from EVOO can induce anti-cancer effects in breast cancer cells
	Fini, L. [65]	2008	Evaluation of anti-cancer effects of EVOO phenolic extracts in cells lines for two EVOOs. (1). EVOO (A) pinoresinol as main phenol (2). EVOO (B) oleocanthal as main phenol	EVOO (A) has powerful chemopreventive actions and upregulates the ATM-p53 cascade	EVOO (A) inhibits cell proliferation in a dependent manner. The comparison between effects of EVOO (A) and (B) demonstrates significant powerful effects of EVOO (A) respect to EVOO (B) *p* < 0.0001
	Zambonin, L. [33]	2012	Evaluation of the antioxidant activity of phenolic acids in HEL cells	Proapoptotic effects in leukemia cells	In HEL cells: - Induce apoptosis - Increase caspases 3,8,9 activity - Increase ratio Bax/Bcl2 - Reduce Akt activation
	Incani, A. [38]	2016	Evaluation of two monovarietal EVOO phenolic extracts (Bosana and Nera) on Caco-2 cells	Modulation of enterocyte response to oxidative and inflammatory stimuli after absorption of EVOO	- Protection of Caco-2 cell monolayers against TBH and oxysterols oxidative injury - ROS production inversely correlated to decrease of GSH levels - Attenuation of TBH-induced oxidative damage (Bosana type the most active)
Animal	Priora, R. [61]	2008	Randomized study in 6 groups for different treatments (10 rats x group). They tested 3 types of oil characterized by different MPC concentration: refined olive oil with trace MPC (control), low-MPC EVOO, and high-MPC EVOO	Effect of EVOO in relation to MPC on platelet aggregation and plasma concentration of Hcy redox form	- MPC of EVOO inhibits platelet aggregation and decreases the concentration of Hcy redox form
Humans	Keys, A. [16]	1986	Study among 15 different cohorts (*n* = 11.579 healthy males) on mortality from all causes, follow-up period 15 years	All cause and coronary disease death during 15-year follow-up was significantly lower in cohorts with olive oil as main fat	- N.A.
	De Lorgeril, M. [25]	1994	MD alpha-linolenic acid rich vs. prudent diet in secondary prevention of CHD patients	Secondary prevention of coronary events and deaths	- Coronary events: - R.R. 0.27 (95% CI, 0.12–0.59) - *p* = 0.001 - Death: - R.R. 0.30 (95% CI, 0.11–0.82) - *p* = 0.082
	Visioli, F. [35]	2000	Six male volunteers 50 mL of olive oil samples accompanied by 40 g of bread, four times	Olive oil phenolics are dose-dependently absorbed in humans	- N.A.
	Riboli, E. [68]	2002	Multicenter prospective cohort study on 521.000 subjects investigation on the relationship between nutrition and cancer	Evaluation of the possible correlation between the incidence of cancer and nutrition	- Data collected indicates that the MD is the most effective food model in cancer prevention
	Salvini, S. [42]	2006	Randomized Cross over trial 10 postmenopausal women about the effect of high-phenol EVOO vs. low-phenol EVOO on oxidative DNA damage	Two types of olive oil were assumed for 8 weeks (50 g/day) and were tested in peripheral blood lymphocytes	- Oxidative DNA damage during assumption of high-phenol EVOO was 30% lower respect to mean values observed during low-phenol EVOO consumption (*p* = 0.02)
	Covas, M.I. [43]	2006	Evaluated, in 200 healthy male volunteers, the effects of polyphenol content in olive oil on oxidative lipid damage and plasma lipid levels	Crossover study, enrolled subjects assumed randomly 3 types of olive oils daily administration (25 mL/day). One type was low-phenols (2.7 mg/kg of olive oil), medium-phenols (164 mg/kg), or high-phenols (366 mg/kg) content. Intervention periods were 3 weeks.	- Values of oxidative stress biomarkers were inversely related to phenolic content
	Masala, G. [72]	2007	Evaluation of dietary patterns on overall mortality in Italian elderly population (aged > 60 years)	“Olive oil and salad” type is inversely associated with all-cause mortality. While the pasta and meat pattern have an increased mortality for all causes.	- All-cause mortality was reduced by about 50% in the highest quartile of the “Olive Oil and Salad” model. - *p* = 0.008
	De Lorenzo, A. [22]	2010	IMD and IMOD vs. usual diet in patients with CKD stage II–III	Effect of diet treatment on laboratory and body composition parameters	- Reduction of: - Hcy (*p* = 0.0116) - *p* (*p* < 0.001) - Microalbuminuria (*p* = 0.0086) - hs-CRP (*p* < 0.05) - FM (kg), FM (%), (*p* = <0.001)
	Bendinelli, B. [73]	2011	Association between fruit, vegetable, and olive oil consumption and the incidence of CHD in Italian women	8-year follow-up in which the possible relationships between dietary habits, lifestyle, anthropometric measures, and the development of CHD major events were evaluated.	- Reverse association between consumption of leafy vegetables and olive oil and risk of developing CHD. Leaf vegetables: H.R. 0.54 (95% CI 0.33–0.90, *p* = 0.03 Olive oil: H.R. 0.56 (95% CI 0.31–0.99, *p* = 0.04)
	Perez-Herrera, A. [44]	2012	Study randomized crossover of 20 obese subjects that received four breakfasts constituted by milk and muffin prepared with one of four different oils: virgin olive oil, sunflower oil, mixture seeds oil with added dimethylpolyxiloxane, or natural antioxidants from olive mill wasterwater alperujo	Evaluations of postprandial inflammatory status in 20 obese subjects by the activation of nuclear NF-kB, the cytoplasmatic concentration of NF-kB inhibitor, the mRNA levels of NF-kB subunits and activators, inflammatory molecules, and LPS levels	- Virgin olive oil and olive mill wasterwater alperujo reduced NF-kB activation, increased NF-kB inhibitor, and decreased LPS plasma concentration - Seed oil increases mRNA expression of NF-kB subunit, inflammatory molecules, and LPS
	Di Daniele, N. [23]	2014	IMD and IMOD in patients with CKD stage II–III vs. low-protein diet according to MTHFR genotypes	Effect of diet treatment on laboratory and body composition parameters	- Reduction of Hcy in T (+) genotypes: -IMD: 3.08 mol/L, 95% CI, 4.94–1.23), *p* = 0.001 -IMOD: 9.18 mol/L, 95% CI, 11.04–7.33), *p* < 0.001
	Agrawal, K. [60]	2017	Double-blind, randomized controlled crossover study on 9 healthy subjects. They assumed 40 mL/week of tree different phenolic content EVOO.	Evaluation of EVOO assumption on inhibition of platelet aggregation pre and 2 h post-EVOO intake	- Decline of Pmax is related to oleocanthal intake (*r* = 0.56 *p* = 0.002)
	Estruch, R. [24]	2018	Mediterranean Diet supplements with EVOO or nuts vs. reduced-fat diet in 7447 Spanish subjects	Major CV events	- MD+EVOO - H. R. 0.69 (95% CI, 0.53–0.91) - MD+NUTS - H.R. 0.72 (95% CI, 0.54–0.95)

Akt, protein kinase B; ATM, ataxia–telangectasia mutated; Bax, (Bcl-2)-associated X protein; Bcl-2, B-cell lymphoma protein 2; Caco-2, heterogeneous human epithelial colorectal adenocarcinoma cell lines; CHD, coronary heart disease; CKD, chronic kidney disease; COX-1, cyclooxygenase-1; COX-2, cyclooxygenase-2; CV, cardiovascular; EVOO, extra virgin olive oil; FASN, inhibitors of fatty acid synthase; FM, fat mass; GSH, glutathione; Hcy, homocysteine; HEL, human erythroleukemia cell lines; HER2, receptor tyrosine-protein kinase erbB-2; hs-CRP, high sensitivity-C reactive protein; HT, hydroxytyrosol; HUVEC, human umbilical vein endothelial cell lines; HVA, homovanillyl alcohol; ICAM-1, intercellular adhesion molecule 1; IMD, Italian Mediterranean diet; IMOD, Italian Mediterranean organic diet; LDL, low-density lipoprotein; LPS, lipopolysaccharide; MD, Mediterranean diet; MDM, monocytes-derived macrophages; MPC-OOE, minor polar compounds olive oil extract; MPC, minor polar compounds; MTHFR, methylene tetrahydrofolate reductase; NF-kB, nuclear factor kappa-light-chain-enhancer of activated B cells; OleA, oleuropein aglycone; P, phosphorus; Pmax, maximum platelet aggregation; ROS, reactive oxygen species; SKBR3, human breast cancer cell lines; TBH, tert-butyl hydroperoxide; VCAM-1, vascular cell adhesion molecule 1; VOO, virgin olive oil.

**Table 3:** Biological activity *of Olea europaea* L. by-products.

By-Products of EVOO Process					
Type of Study	Reference	Year	Type of Intervention	Primary Outcome	*p*-Value for Primary Endpoint
In vitro cell models	Obied, H.K. [88]	2007	Olive mill waste waters tested against *Staphylococcus aureus*, *Bacillus subtilis*, *Escherichia coli*, *Pseudomonas aeruginosa*, *Candida albicans*, *Aspergillus niger.*	Antibacterial activity against *S. aureus*, *B. subtilis*, *E. coli*, and *P. aeruginosa*	At lower concentrations, the extracts exhibited differential antibacterial action, but at 5 mg/disc extracts were active against all the challenge bacteria
	Schaffer, S. [87]	2010	OMWW extracts and HT were evaluated for their cytoprotective effects in an in vitro model of neuronal-like PC12 cells	Cytoprotective effects in PC12 cells subjected to oxidative or nitrosative stress by adding either ferrous iron or sodium nitroprusside to the cell culture medium for 18 h	Incubating PC12 cells with wastewater extract protect from nitrosative stress. The extract was able to maintain ATP levels but not MMP.
	Bernini, R. [93]	2017	Lipophilic fractions from Olea by-products were tested on human colon cancer cell line HCT8-β8 engineered to overexpress estrogen receptor β (ERβ)	Antiproliferative effect	HT and lipophilic fractions significantly reduced the proliferation of HCT8-β8-expressing cells in a concentration-dependent manner. HT oleate showed the greater effect.
	Plastina, P. [95]	2019	Phenolic extracts from OMWW were tested for their ability to reduce NO production by LPS-stimulated RAW-264.7 macrophages	Anti-inflammatory activity	HT stearate and HT oleate decrease NO production in a concentration-dependent manner
Humans	Visioli, F. [96]	2009	OMWW extracts were tested on human volunteers 1 h after ingestion	Plasma antioxidant capacity and total reduced glutathione	No difference in plasma antioxidant capacity; a significant increase in total plasma glutathione concentration

ATP, adenosine triphosphate; ERβ, estrogen receptor beta; HT, hydroxytyrosol; LPS, lipopolysaccharide; MMP, mitochondrial membrane potential; NO, nitric oxide; OMWW, olive mill wastewater; RAW-264.7, Abelson murine leukemia virus transformed.

**Table 4:** Biological activity olive leaf extracts.

Olive Leaf Extracts					
Type of Study	Reference	Year	Type of Intervention	Primary Outcome	*p*-Value for Primary Endpoint
In vitro cell models	Andrikopoulos, N.K. [100]	2002	Effects against copper ion-induced low-density lipoprotein (LDL) oxidation	LDL mean protection activity	Quercetin, luteolin, and rutin, activities 46.8%, 49.5%, and 53.7% MP, respectively, comparable to oleuropein the 49.0% MP
	Sudjana, A.N. [107]	2009	Antimicrobical activity	Role in regulating the composition of the gastric flora	Specific activity, in reducing levels of *H. pylori* and *C. jejuni*.
	Rigacci, S. [109,110]	2010 2011	Effects on amylin and peptide aggregation and cytotoxicity	Hindering amylin and Aβ-peptide aggregation, preventing their cytotoxicity	Increased viability of β-pancreatic and neuroblastoma cells decreasing caspase-3 activity
	Rigacci, S. [111]	2015	Neuroprotection effect	Autophagy induction both in vitro in neuronal cells and in in vivo Aβ model deposition (TgCRND8 mice) by Ca2+/CaMKKβ/AMPK/mTOR axis	Cytosolic Ca^2+^ increase activates CaMKKβ and pAMPK concomitant with increased beclin1/LC3II and decreased phospho-mTOR and phospho-p70S6K expression
	Papachristodoulou, A. [105]	2016	Anticancer effect and adjuvant to antitumoral therapies	Lowering of the cytotoxic dose in doxorubicin to obtain the same antiproliferative effect in prostate cancer	Remarkable induction of autophagy correlated to significant metabolite alterations
	Luccarini, I. [112]	2016	Neuroprotection effect	Counteracting neuronal damage through modulation of the PARP1–SIRT1 interplay both in neuronal cells and in TgCRND8 mice	In vitro reduction of PARP1 activation and paralleled overexpression of Sirtuin1. In vivo, (in addition to above reported effects), a decrease of NF-kB and of the pro-apoptotic marker p53 expression
	Miceli, C. [108]	2018	Cardioprotective effect	Cardioprotection on MAO-A overexpressed cardiomyocytes by restoring the defective autophagic flux due to oxidative stress	Reduction of MAO-induced cardiotoxicity through MTT. Autophagy induction by TFEB nuclear translocation.
	Ruzzolini, J. [118]	2018	Anticancer effect and adjuvant to antitumoral therapies	Reduction of viability of BRAF melanoma cells. Enhanced effects with chemotherapic drugs (dacarbazina and everolimus) at no toxic dose.	High dose induced cell death by apoptosis, while no toxic dose affected viability through the inhibition of phosphorylation of AKT and the S6 pathway
	Margheri, F.M.B. [114]	2019	Effect on tumor microenvironment	Anti-angiogenic activity in senescence-associated-secretory-phenotype (SASP) fibroblast cultured media	Decrease of pro-angiogenic factors release in SASP fibroblasts cultured media and inhibition of cell-dependent invasion and of capillary-like structure formation of endothelial cells exposed to the above media
Animals	Jemai, H. [102]	2009	Effects in alloxan-diabetic rats	Hypoglycemic and antioxidant activity	
	Andreadou, I. [119]	2014	Effect on chronic doxorubicin induced cardiomyopathy	Prevention of the structural, functional, and histopathological cardiac effects	Activation of AMPK and suppression of iNOS. Reduction of pro-apoptotic mediators and modulation of myocardial metabolism.
	Rigacci, S. [111]	2015	See above		
	Luccarini, I. [112]	2016	See above		
	Janahmadi, Z. [101]	2017	Cardioprotection in rats with heart failure	Antioxidative and anti-inflammatory effects	Increase of SV, EF, FS, and CO (*p* < 0.05), serum SOD and GRx. Reduction of serum MDA, IL-1β or TNF-α (*p* < 0.05).
Humans	De Bock, M. [104]	2013	46 Participants (aged 46.465.5 years and BMI 28.062.0 kg/m^2^) were randomized to receive capsules with olive leaf extract (OLE) or placebo for 12 weeks	Improvement in insulin sensitivity and β-pancreatic cell secretory capacity	Insulin sensitivity (*p* = 0.024). β-pancreatic cell responsiveness (*p* = 0.013).
	Carnevale, R. [103]	2018	Twenty healthy subjects were randomized to receive 20 mg oleuropein or 20 mg placebo before lunch	Improvement in postprandial glycemic profile	Lower blood glucose, DPP-4 activity, and higher insulin and glucagon-like peptide-1 vs. placebo

AKT, protein kinase B; AMPK, 5′ adenosine monophosphate-activated protein kinase; BMI, body mass index; BRAF, B-Raf proto-oncogene; CaMKKβ, Ca2+/calmodulin-dependent protein kinase kinase β; CO, cardiac output; DPP-4, dipeptidyl peptidase-4; EF, ejection fraction; FS, fractional shortening; GRx, glutathione reductase; HT, Hydroxytyrosol; IL-1β, interleukin-1β; iNOS, nitric oxide inducible isoform; LDL, low-density lipoprotein; MAO-A, monoamine oxidase A; MDA, malondialdehyde; MP, mean protection; mTOR, mammalian target of rapamycin; MTT assay, 3-(4,5-dimethylthiazol-2-yl)-2,5-diphenyltetrazolium bromide; NF-KB, nuclear factor kappa-light-chain-enhancer of activated B cells; OLE, oleuropein; ORAC, oxygen radical absorbance capacity; PARP1, poly[ADP-ribose] polymerase 1; SASP, senescence associated secretory phenotype; SIRT1, NAD-dependent deacetylase sirtuin-1; SV, stroke volume; TEAC, Trolox equivalent antioxidant capacity; TFEB, transcription factor EB; TNF-α, tumor necrosis factor-α.

## Abbreviation List

CKD	Chronic kidney disease
COX-1	Ciclooxygenase-1
COX-2	Ciclooxygenase-2
CV	Cardiovascular
CVD	Cardiovascular disease
DXR	Doxorubicin
EFSA	European Food Safety Authority
EPIC	European Prospective Investigation into Cancer and Nutrition
EPICOR	Long-term follow-up of antithrombotic management patterns on acute coronary syndrome patients
EU	European Union
EVOO	Extra-virgin olive oil
Hcy	Homocysteine
HDL	High density lipoproteins
HPLC-DAD	High performance liquid chromatography-diode array detector
HPLC-MS	High performance liquid chromatography-mass detector
HT	Hydroxytyrosol
HUVEC	Human endothelial cells
IMD	Italian Mediterranean diet
IMOD	Italian Mediterranean organic diet
IOC	International Olive Council
K-DOQI	Kidney-Disease Outcomes Quality Initiative
LDL	Low density lipoprotein
LOX-1	Lectin-like oxidized LDL receptor-1
MD	Mediterranean diet
MDM	Monocyte- derived macrophages
MFA	Membrane Filtration Absorption
MTHFR	Methylenetetrahydrofolate reductase
NCD	Chronic non-communicable disease
NF-κB	Nuclear factor kappa-light-chain-enhancer of activated B cells
NO	Nitric oxide
OLC	Oleocanthal
OLE	Oleuropein
OO	Olive oil
OxLDL	Oxidized low density lipoprotein
PMA	Phorbol-myristate acetate
PPAR-γ	Peroxisome proliferator-activated receptor gamma
RCT	Randomized controlled trial
ROS	Reactive oxygen species
SASP	Senescence-associated-secretory-phenotype
SCSCD	Seven Country Study of Cardiovascular Disease
SOP	Stoned Olive Pomace
TRPA1	Transient receptor potential channel, subfamily A, member 1
Tyr	Tyrosol
UNESCO	United Nations Educational Scientific and Cultural Organization
